# Neurofibromatosis Type 1 Complicated by Atypical Coarctation of the Thoracic Aorta

**DOI:** 10.1155/2013/458543

**Published:** 2013-03-04

**Authors:** Masato Kimura, Shuhei Kakizaki, Kengo Kawano, Shinichi Sato, Shigeo Kure

**Affiliations:** Department of Pediatrics, Tohoku University Graduate School of Medicine, 1-1 Seiryo-Cho, Aoba-ku, Sendai, Miyagi 980-8574, Japan

## Abstract

Neurofibromatosis type 1 (NF1) is a relatively common autosomal dominant genetic disorder with a prevalence of 1 in 3,000 (0.03%) at birth. Clinical features are café-au-lait macules, intertriginous freckling, dermal neurofibroma, iris hamartoma (Lisch nodules), and learning disability. NF1 vasculopathy is a serious but underrecognized complication involving the cerebrovascular and cardiovascular systems. The incidence of hypertension in patients with NF1 is around 1% and is associated mainly with renal artery stenosis in children. Only a few cases of thoracic aortic coarctation in association with hypertension and neurofibromatosis have been reported. Here we describe the case of a 4-year-old girl who presented with NF1 and hypertension due to atypical coarctation of the thoracic aorta. The diagnosis of coarctation of the thoracic aorta at the Th5-to-Th6 level was made following catheterization with a pressure gradient of 40 mmHg. The patient underwent surgery comprising resection of the coarctation of the thoracic aorta and graft interposition. On the basis of our findings, annual assessment of blood pressure is advised for patients with NF1.

## 1. Introduction

Neurofibromatosis type 1 (NF1; OMIM #162200) is a dominantly inherited multisystem genetic disorder with a prevalence of approximately 1 in 3,000 (0.03%) at birth [1]. The disease phenotype is variable, with typical characteristics including café-au-lait spots on the skin, axillary and inguinal freckling, multiple neurofibroma, iris hamartoma (Lisch nodules), and learning disability. NF1 vasculopathy is a potentially serious but less well-known manifestation of this multisystem genetic disorder, which can produce renal artery stenosis, coarctation of the aorta, and other vascular lesions [2, 3]. The incidence of hypertension in patients with NF1 is around 1% and is associated most commonly with renal artery stenosis in children and pheochromocytoma in adults. To date, only a few cases of thoracic aortic coarctation in association with hypertension and neurofibromatosis have been promoted [4]. Here we report the case of a 4-year-old girl with hypertension and atypical thoracic coarctation who underwent graft interposition.

## 2. Case Report

**Figure 1:**
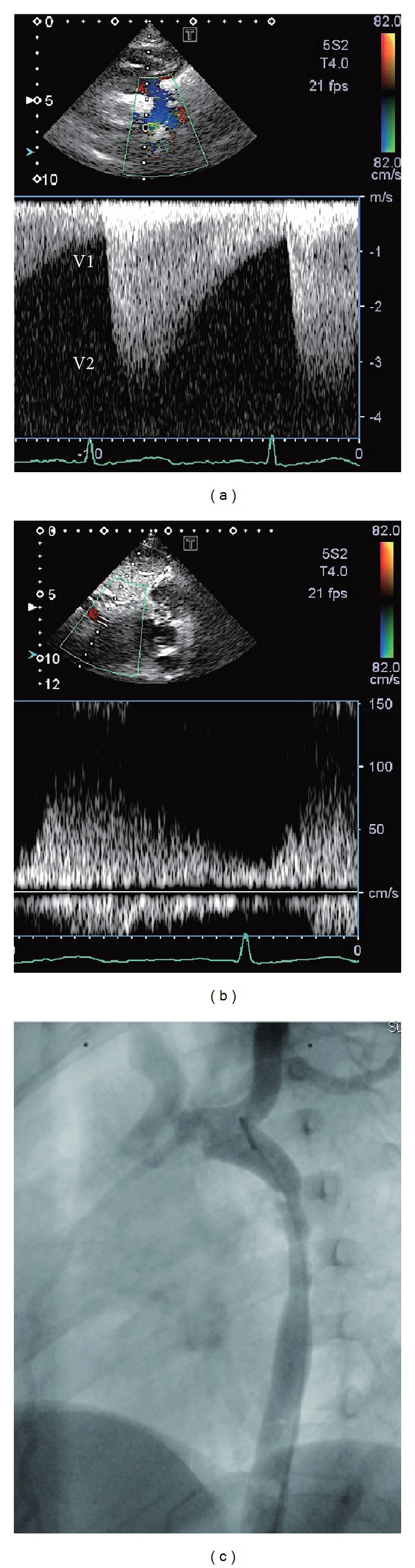
(a) Continuous wave Doppler flow pattern across the coarcted segment, showing systolic flow velocity of 3.4 m/s. V1: flow proximal to the coarctation. V2: flow distal to the coarctation. (b) Pulsed Doppler evaluation of the abdominal aorta demonstrating the decrease in the acceleration and deceleration slope. (c) Aortogram of the descending aorta (left anterior oblique view) showing a 5 cm long hourglass-shaped thoracic coarctation at the Th5-to-Th6 level with the narrowest section having a diameter of 4 mm as well as a large internal thoracic artery.

**Figure 2:**
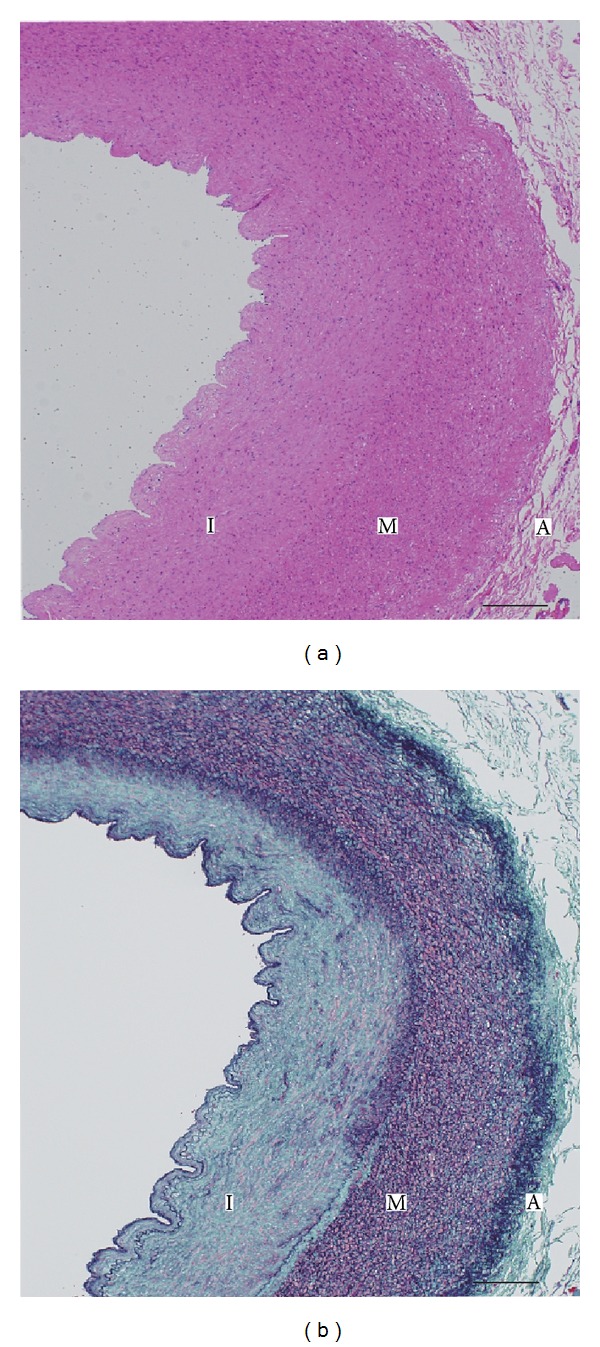
Photomicrograph of thoracic aortic coarctation. Cross-sections were stained with hematoxylin-eosin (a) and Elastica-Masson (b). In panel (b), intimal proliferation (I) of collagen tissue (green) and smooth muscle (pink) is shown. Elastic fiber (purple) is decreased and substituted by collagen fiber in the outer media (M). I: tunica intima; M: tunica media; A: tunica adventitia. Scale bar: 200 *μ*m.

A 4-year-old girl was referred to our hospital with hypertension and suspected aortic coarctation. She was a full-term normal delivery without any antenatal or postnatal complications and a birth weight of 2.5 kg. At 5 months of age, heart murmur due to a small ventricular septal defect was diagnosed at a referred hospital. At the age of four, a regular medical examination revealed elevated arterial blood pressure of 140/90 mmHg at the upper arm and weak femoral arterial pulse. She had no complaint of headache and no history of any type of seizure. In addition, her family history was not suggestive of neurofibromatosis. On physical examination at our hospital, she weighed 22 kg and her height was 106 cm. She had a strong pulse in the upper extremities and a weak pulse in the lower extremities, and posterior cardiac auscultation revealed systolic murmur of grade 4 (Levine scale) in the interscapular area. She also had more than 6 cafe-au-lait spots larger than 5 mm, and ophthalmological examination revealed multiple Lisch nodules of the iris. Brain magnetic resonance imaging uncovered multiple focal areas of signal intensity in the globus pallidus, thalamus, hippocampus, and dental nucleus on T2-weighted images. Chest radiography showed normal pulmonary vascularity and no cardiomegaly (cardiothoracic ratio, 50%), and the results of electrocardiography did not fulfill the criteria for left ventricular hypertrophy. Two-dimensional echocardiography showed increased left ventricular posterior wall thickness (6.8 mm; 120% of normal) with normal left ventricular systolic function. Pulsed Doppler echocardiography findings of the suprasternal notch and descending aorta indicated characteristic flow patterns of significant aortic coarctation (Figures 1(a) and 1(b)). No abnormal intracardiac shunts were detected. Cardiac catheterization revealed ascending aorta pressure of 140/90 mmHg (mean, 120 mmHg) and distal thoracic aorta pressure of 100/80 mmHg (mean, 90 mmHg). Aortography of the descending aorta (left anterior oblique view) showed a 5 cm long hourglass-shaped thoracic coarctation at the Th5-to-Th6 level with the narrowest section having a diameter of 4 mm, as well as a large internal thoracic artery (Figure 1(c)). Cardiac surgery was performed, and the coarctation was excised and replaced with a 14 mm ePTFE graft without complications. Pathologic examination of the specimen showed the accumulation of smooth muscle cells and collagen tissue in the intimal layer of the thoracic aorta, resulting in hyperplasia that narrowed the lumen (Figure 2). The patient was discharged in good hemodynamic condition without a difference in blood pressure between the arms and lower extremities. During the postoperative period, angiotensin converting enzyme inhibitor therapy was initiated because of persistent postoperative hypertension.

## 3. Discussion

NF1 (OMIM #162200) is an autosomal dominant multisystem genetic disorder caused by a mutation in the NF1 gene on chromosome 17 (17q11.2). The clinical diagnostic criteria for NF1 were established in 1988 by the National Institute of Health and are satisfied when two of the following symptoms are met [5]: café-au-lait spots on the skin (≥6 spots >5 mm before puberty or >15 mm after puberty), intertriginous freckling (axillae, groin, and neck), dermal neurofibroma (≥2), optic pathway glioma, iris hamartoma (Lisch nodules), skeletal dysplasia (tibial dysplasia or sphenoid wing dysplasia), and a positive family history of NF1 affecting a first-degree relative. Café-au-lait spots, which are present in all patients, may be the only feature apparent in some patients, especially infants and children. Young children with multiple café-au-lait spots and no other NF1 features whose parents do not show signs of NF1 are recommended to undergo ophthalmologic examination. Lisch nodules are not considered an ophthalmologic complication, while multiple Lisch nodules, unlike café-au-lait spots and neurofibromas, are specific to neurofibromatosis [5]. Moreover, symptomatic optic pathway glioma in individuals with NF1 usually presents before 8 years of age with loss of visual acuity or proptosis, but these tumors may not become symptomatic until later in childhood or even adulthood. 

 NF1 is associated with vasculopathy, which is a significant but underrecognized complication that affects multiple sites, including thoracic, abdominal, renal, and intracranial vessels. Vasculopathy may lead to occlusions and aneurysms of any known size at any location in the body, and when involving major arteries or arteries of the heart or brain, vasculopathy can have serious or even fatal consequences [6, 7]. NF1 vasculopathy can produce renal artery stenosis, coarctation of the aorta, and other vascular lesions associated with hypertension. Renal artery stenosis has been recognized as the most common form of secondary hypertension in children with neurofibromatosis. However, there are only a few reports of thoracic aortic coarctation in association with hypertension and neurofibromatosis [4]. It is noteworthy that not all NF1 vasculopathy is congenital, but at least some NF1 vasculopathy progresses after birth [8]. Similar to the findings presented in previous reports [7, 9], the gross and microscopic pathologic findings in our patient reflected the aortic coarctations found in patients with neurofibromatosis, including intimal hyperplasia of proliferating smooth muscle cells and luminal narrowing (Figure 2).

 Neurofibromin, the protein product of NF1, is normally expressed in the epithelial and smooth muscle cells of blood vessels, can regulate cell growth through Ras regulation, and is less likely to be involved in pathogenesis, but its precise mechanisms are poorly understood [10]. It has been reported in animal models [11] and in studies of human endothelial cells [12] that the loss of neurofibromin expression in endothelial cells may cause proliferation of vascular smooth muscle cells. 

 In summary, a 4-year-old girl underwent successful cardiac surgery for hypoplasia of the thoracic aorta after presenting with NF1 and severe hypertension. Taken together, ophthalmologic examination is recommended for young children with multiple café-au-lait spots. Furthermore, it is important to note that appropriate cardiac examination and blood pressure monitoring should be part of the routine care provided to NF1 patients.
